# Directional preparation of anticoagulant-active sulfated polysaccharides from *Enteromorpha prolifera* using artificial neural networks

**DOI:** 10.1038/s41598-018-21556-x

**Published:** 2018-02-15

**Authors:** Jiefen Cui, Yinping Li, Shixin Wang, Yongzhou Chi, Hueymin Hwang, Peng Wang

**Affiliations:** 1 College of Food Science and Engineering, Ocean University of China, Qingdao, 266003 PR China; 2 College of Marine Science and Biological Engineering, Qingdao University of Science & Technology, Qingdao, 266042 PR China; 3 Marine Biomedical Research Institute of Qingdao, Qingdao, 266071 PR China; 4 Biology Department, Jackson State University, Jackson, Mississippi 39217 USA

## Abstract

The sulfated polysaccharides from *Enteromorpha prolifera* (PE) are a potential source of anticoagulant agents. In this study, the PE was degraded by specific degradase and five hydrolysis products with different molecular weights were prepared. The product of 206 kDa is a kind of high rhamnose-containing polysaccharide with sulfate ester (34.29%). It could effectively prolong the activated partial thromboplastin time (APTT), which indicated inhibition of the intrinsic coagulation pathway. The artificial neural network (ANN) was built to realize the directional preparation of anticoagulant-active polysaccharides. Based on monitoring glucose concentration on-line, a visualization system of enzymatic hydrolysis was developed to simplify the operation of ANN. The model could be further applied to predict molecular weights of polysaccharides that possess diverse biological activities.

## Introduction

*Enteromorpha prolifera*, one of the most common green algae, is widely distributed along the intertidal zone of coastal areas^1^. It is regarded as an abundant source of carbohydrates, protein, minerals, crude fiber, and vitamins^2,3^. Polysaccharides from *E*. *prolifera* (PE) possess a wide range of pharmacological properties which can be evidenced by their antitumor, antioxidant, and anticoagulant activities^4,5^. Among these activities, the anticoagulant property is becoming a hot topic in natural products research.

The molecular weight and sulfate esters are the most important parameters for determining anticoagulant activities^6^. However, the high molecular weight and high viscosity of polysaccharides would restrict their pharmaceutical applications. Degradation of PE to low molecular weight polysaccharides might result in more exposure of their active moieties^7^. In recent years research on enzymatic hydrolysis has become popular because of its advantages such as high efficiency and environmental compatibility^8^. However, enzymatic hydrolysis will lead to the formation of complex mixture of molecules which makes the assessment of individual species unfeasible^9^. Consequently, it is essential to establish a method with less experimental effort to conveniently assess the process of the enzymatic hydrolysis reaction.

Artificial neural network (ANN), a non-parametric model, utilizes interconnected mathematical neurons to form a network that could model complex functional relationships^10^. ANN could discover behaviors and patterns from a finite set of data (called the “training set” or “training patterns”). If an adequate training set is provided, the ANN is able to generalize the knowledge acquired during the “learning” process, responding adequately to new values not contained in the “training set”^11^. The ANN is an effective and applicable method to predict the relationship between dependent and independent parameters while the mathematical formulation is unavailable^12^.

In this study, PE with high molecular weight was extracted from *E*. *prolifera* with hot water. The PE was degraded to five fragments with low molecular weight. The anticoagulant activities of the six sulfated polysaccharides were investigated. The ANN was developed to predict molecular weights of sulfated polysaccharides that exhibited potent anticoagulant activity.

## Results and Discussion

### Chemical characterizations

As shown in Table 1, PE consisted of four monosaccharides, including rhamnose (41.42%), glucose (32.53%), xylose (13.78%), and glucuronic acid (11.48%). Shi *et al*.^13^ reported a polysaccharide from *E*. *prolifera* (Zhejiang, China), which was composed of rhamnose (67.8%), glucose (18.6%), xylose (7.7%), galactose (4.0%), and mannose (1.4%). Kim *et al*.^14^ prepared three polysaccharides from *E*. *prolifera* (Wando, Korea), and they were composed of rhamnose (57.1–87.6%), glucose (3.6–39.1%), and xylose (2.4–8.8%). Altogether, rhamnose was the major component of the polysaccharides in *E*. *prolifera*.

**Table 1:** The chemical characteristics of the samples.

Samples	Sulfate (%)	Rhamnose (%)	Glucose (%)	Xylose (%)	Glucuronic (%)	Mw (kDa)
PE	23.96	41.42	32.53	13.78	11.48	1012
PE1	26.34	45.67	9.65	16.75	20.12	602
PE2	28.98	47.68	12.92	17.94	19.74	422
PE3	34.29	53.09	21.42	9.96	15.52	206
PE4	38.21	61.59	3.54	22.43	12.48	104
PE5	40.42	67.35	2.93	12.58	17.15	52

As can be seen from Table 1, the sulfate ester content of PE was 23.96%. Li *et al*.^15^ reported that content of sulfate ester in the polysaccharide from *E*. *prolifera* was 23.17%. Zhang, Wang, Mo, and Qi^7^ reported that the polysaccharide from *E*. *linza* contained 22.4% sulfate ester. Qi *et al*.^5^ prepared the polysaccharide from *E*. *clathrate* in which the sulfate ester content was 31%. Consequently, polysaccharides from green alga *Enteromorpha* were rich in sulfate esters, compared with those from brown alga *Eisenia bicyclis* (13.2%)^16^ and red alga *Furcellaria lumbricalis* (15.2%)^17^. Qiao *et al*.^18^ reported that polysaccharide from *E*. *prolifera* exhibited well-behaved pseudoplastic and thixotropic property. As is shown in Table 1, the molecular weight of PE was 1012 kDa. Polysaccharides with high molecular weights and apparent viscosity are difficult to pass through organizational barriers, which might limit their biological activities^19^.

### Anticoagulant activity assay

We prepared five low molecular weight fractions with the method of enzymatic hydrolysis. The anticoagulant activities of all the samples were determined with the classical coagulation APTT, TT, and PT assays *in vitro* that characterize different stages of the coagulation process. APTT is employed to evaluate the coagulation factors in the intrinsic blood coagulation pathway. PT is used to characterize the extrinsic coagulation factors while an increase in the TT suggests either thrombin inhibition or impaired fibrin^20^. In fact, there was a lack of PT and TT activities in all of the samples.

**Figure 1:**
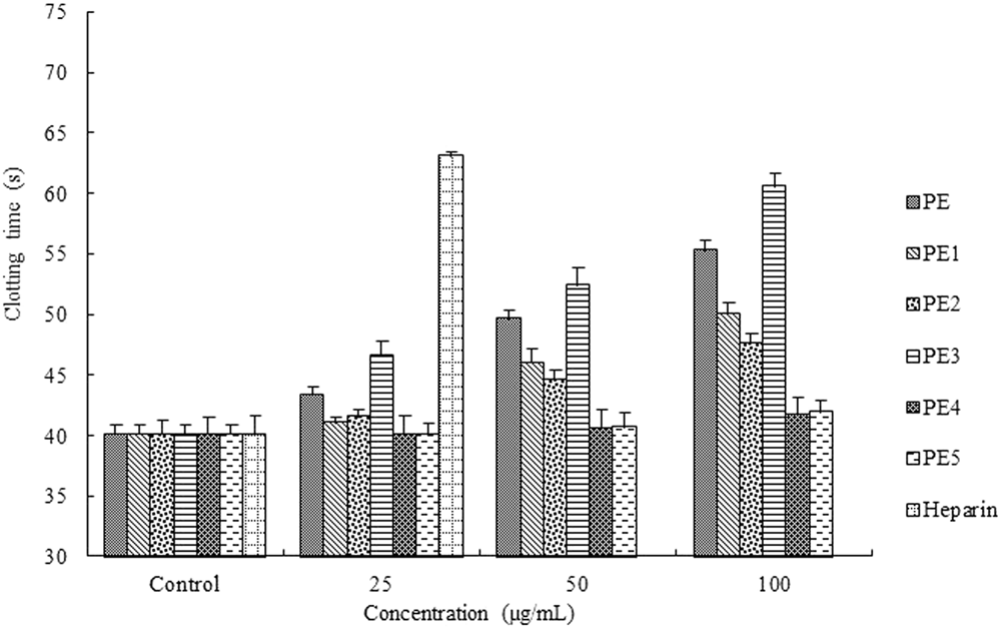
Anticoagulant activity measured by APTT assay.

As seen in Fig. 1, the APTT were prolonged by PE, PE1, PE2, and PE3 in a concentration-dependent manner. The PE3 exhibited the most remarkable APTT activity and the clotting time was 60.56 s at 100 μg/mL. The prolongation of APTT suggests inhibition on the intrinsic coagulation pathway.

**Figure 2:**
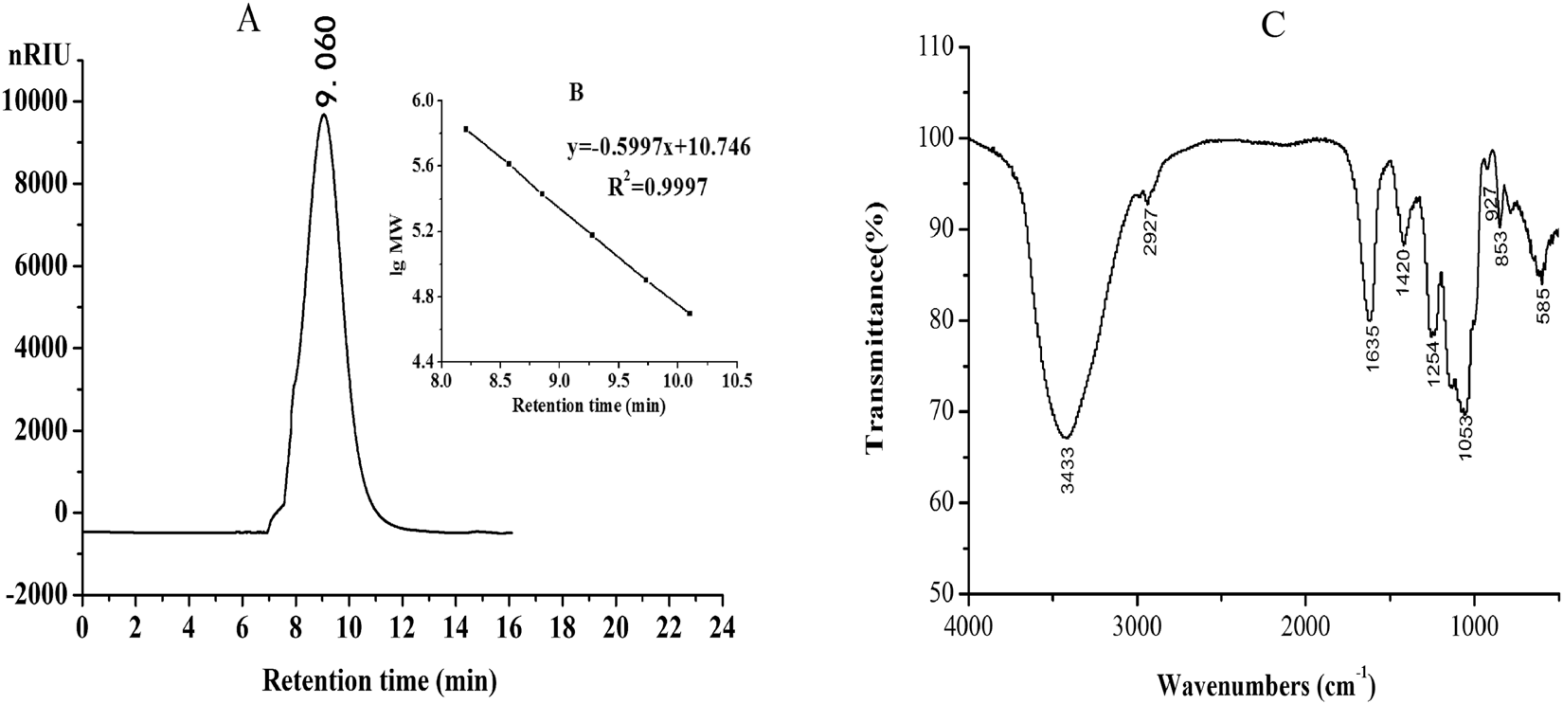
HPGPC chromatogram of PE3 (**A**), Standard curve of molecular weight (**B**), FTIR spectrum of PE3 (**C**).

The HPGPC chromatogram of PE3 is illustrated in Fig. 2(A). The HPGPC chromatogram appeared as a single and symmetrical peak, which demonstrated PE3 was a kind of homogeneous polysaccharide. The FTIR spectrum of PE3 is shown in Fig. 2(C). The absorption bands at 3433 and 2927 cm^−1^ were generated by the stretching vibration of O-H and stretching vibration of C-H, respectively. The bands at 1635 and 1420 cm^−1^ suggested the stretching vibration of C=O and C-O of a carboxyl group, indicating the presence of uronic acid^21^. The bands at 1254 and 853 cm^−1^ were attributed to the stretching vibration of S=O and C-O-S, and indicated the existence of sulfate esters^22^.

The structural characteristics of the sulfated polysaccharides play an important role in the understanding of anticoagulant activity^23^. According to Kamide *et al*.^24^, anticoagulant activity of sulfated cellulose significantly increased with the sulfate ester in the C-2 and C-3 sites, but not in the C-6 site. Li *et al*.^23^ reported an anticoagulant-active polysaccharide from *Monostroma angicava* and sulfate esters substitution occurred at C-3 of (1 → 2)-linked-L-rhamnose residues. Wang *et al*.^25^ prepared three anticoagulant-active polysaccharides from *E*. *linza*, with sulfate esters residing at the C-3 of (1 → 4)-linked- L -rhamnose residues. Our research group reported that the backbone of the polysaccharides from *E*. *prolifera* consisted of D-GlcUAp-α-(1 → 4)-3-sulfate-L-Rhap-β-(1 → 4)-D-Xylp-β-(1 → 4)-3-sulfate-L-Rhap units, with sulphate ester linked on C-3 position of Rha^26^. On the other hand, Wang *et al*.^25^ reported sulfated rhamnose was possible the anticoagulant compound in green algae. The data above suggest that the anticoagulant activity of sulfated polysaccharide was related to sulfate ester position, monosaccharide composition, and glycosidic linkage.

Moreover, the content of sulfate esters also plays an important role in anticoagulant activities. It was reported that anticoagulant activity of the sulfated polysaccharides was partially caused by the strong interaction between the negatively charged sulfate esters and some positively charged peptidic sequences of proteins involved in coagulation process. It is known that Lysine and Arginine residues were present in the heparin-binding site^27^. Therefore, it is possible that the high level of sulfate ester (34.29%) increased the anticoagulant activity of PE3.

The molecular weight of sulfated polysaccharide is another important factor influencing its anticoagulant activity. It was reported that the sulfated polysaccharides from *Monostroma latissimum* required longer chains to achieve the inhibition of thrombin^28^. On the other hand, the spatial arrangement of sulfate ester was reported being important for anticoagulant activity^29^. Therefore, the spatial arrangement of PE3 might be the advantageous conformation and it could promote a better interaction with blood coagulation factors. As can be seen in Fig. 1, PE4 and PE5 with low molecular weights showed no prolongation of APTT. We assumed that some structural change, such as a shift in conformation, caused the extension of enzymatic hydrolysis time.

In conclusion, the potent anticoagulant activity of PE3 depended on the integration of many factors. Realization of accurate preparation of PE3 would enhance its application in pharmaceutical industries.

### Artificial neural network for predicting molecular weight distributions

The ANN is a parallel processing network which determined the complex nonlinear relationships between independent and dependent variables^30^. The back-propagation (BP) network is one of the most widely used ANN for multilayered feed-forward networks^31^. The learning rule of BP network is to use the steepest descent method to continuously adjust the weights and thresholds^32^. In this work, a feed-forward neural network trained with an error BP algorithm was employed in constructing and training the ANN. It is noteworthy that the Levenberg-Marquardt algorithm was applied in the training process. This is a combination of the Grade method and the Gauss-Newton method. It was reported that when such an algorithm is used, time-consuming search can be significantly reduced^33^.

**Figure 3:**
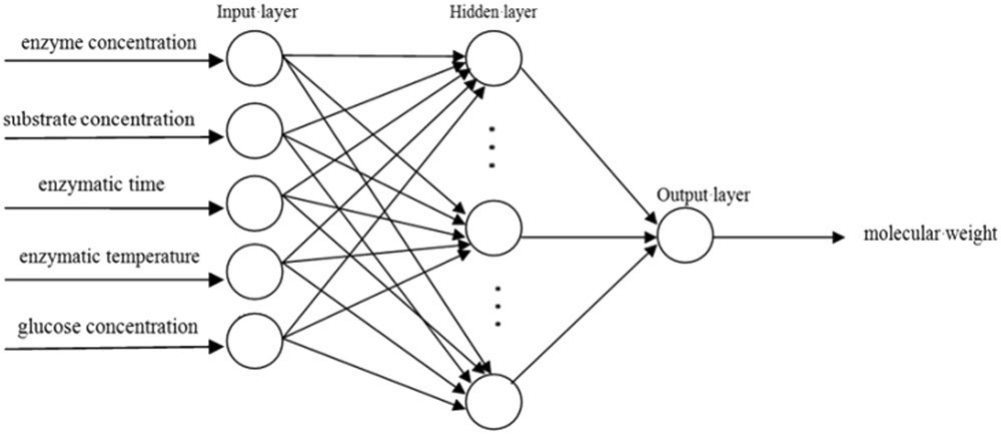
Optimal structure of neural network.

According to Kolmogorov theorem, a three-layer network is sufficient to complete any n-dimensional to m-dimensional nonlinear mapping, thereby setting only one hidden layer structure^34^. In the network, enzyme concentration, substrate concentration, enzymatic temperature, enzymatic time, and glucose concentration were assigned as five input layer nodes while molecular weight was output layer node (Fig. 3). The input layer provides the hidden layer with the sum of the weighted input parameters. These weights are adjusted automatically by the BP algorithm during training.

Different combinations of the transfer functions (logsig, tansig, and purelin) were studied to determine the best combination of two transfer functions that will yield accurate results (Table 2). The performance of these combinations was evaluated on the basis of mean absolute percentage error (MAPE). The MAPE of 0–10%, 10–20%, 20–50%, and >50% represents high prediction accuracy, good prediction, normal accuracy, and bad accuracy, respectively^35^. As can be seen in Table 2, the MAPE of all the combinations is in the range of 0–10%. The tansig and purelin were used as the transfer functions for input and output layer, respectively, because of the minimum MAPE (2.14%).

**Table 2:** Transfer function combinations and their performance.

	Input	Output	MAPE
Transfer function	Tansig	Tansig	8.76%
	Tansig	Logsig	9.35%
	Tansig	Purelin	2.14%
	Purelin	Purelin	5.13%
	Purelin	Tansig	4.48%
	Purelin	Logsig	6.46%
	Logsig	Logsig	8.24%
	Logsig	Tansig	4.93%
	Logsig	Purelin	7.36%

**Figure 4:**
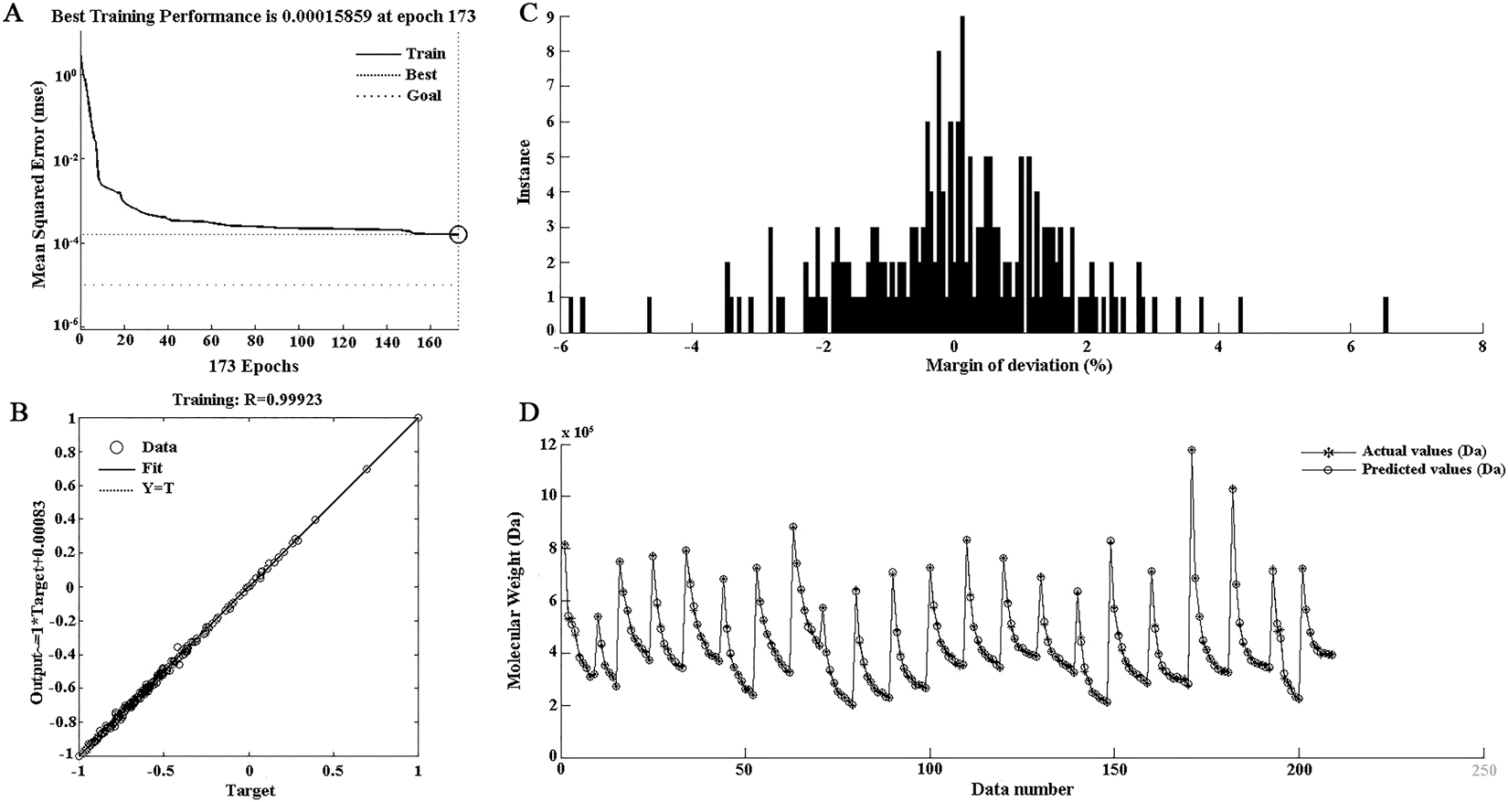
Results of ANN: training performance of ANN (**A**), regression of ANN (**B**), histogram of deviation margin of ANN (**C**), ANN outputs versus experimental data for training data sets (**D**).

The mean square error (MSE) versus the number of repetitions for training is illustrated in Fig. 4(A). In this case, the MSE of training data is equal to 0.00016, after 173 epochs. As shown in Fig. 4(B), the R value is 0.99923. The model fits well with the actual data when R approaches to 1^36^. Histogram of deviation margin for the optimal ANN is presented in Fig. 4(C). It can be seen that deviation distribution is concentrated around zero, which indicates that the ANN has a remarkable accuracy. A comparison between experimental data and outputs of ANN model is shown in Fig. 4(D). Overall, there is a good fitting between the estimated values and the actual values.

**Figure 5:**
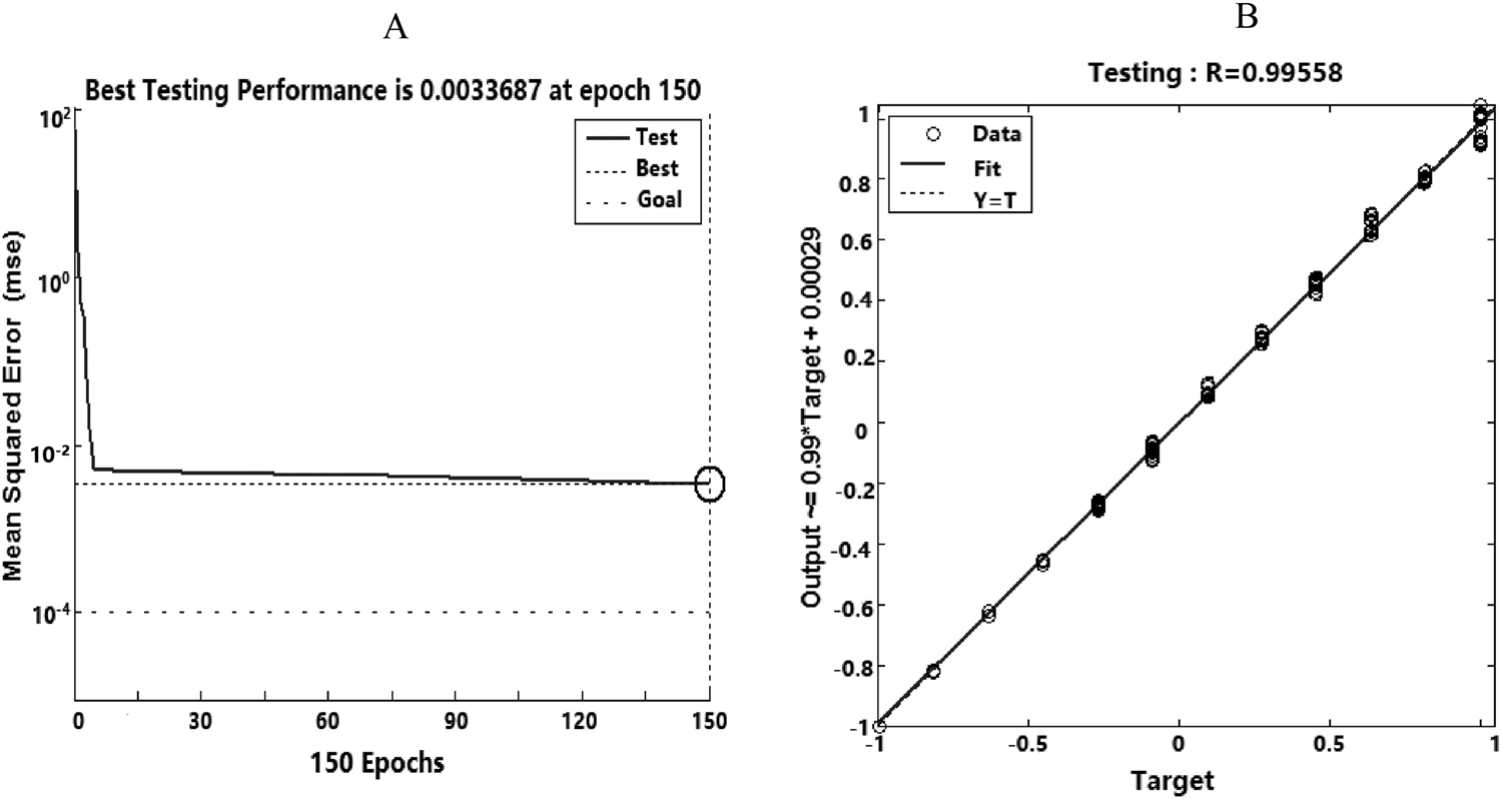
Testing performance (**A**) and regression of ANN (**B**).

The 10% of the testing sets (Table 3) were used for testing the precision of the ANN model. The mean absolute percentage error (MAPE) of molecular weights for testing sets is 1.86% (Table 3). According to Khoshnevisan *et al*.^37^, a MAPE value of less than 10% indicates that the best prediction has been achieved. Therefore, this network could be used with high reliability to estimate the molecular weights. The testing performance and regression of ANN (Fig. 5). As can be seen in Fig. 5(A), testing was stopped after 150 epochs with MSE value of approximately 0.0034. As shown in Fig. 5(B), the R value is 0.99558, which implies good fits between predicted values and the actual values. Therefore, this network could be used with high reliability to estimate the molecular weights.

**Table 3:** Comparison of actual and predicted molecular weight on testing sets.

No.	Enzymatic temperature (°C)	Enzymatic Time (h)	Enzyme dose (U)	Substrate concentration (mg/mL)	Glucose concentration (mg/mL)	Actual molecular weight (kDa)	Predicted molecular weight (kDa)	APE (%)
1	25	5	8.10	4	0.33	417.31	422.04	1.13
2	25	9	8.10	4	0.36	329.91	324.8	1.55
3	25	1	12.15	6	0.27	710.67	706.96	0.52
4	25	4	12.15	6	0.68	388.68	376.18	3.22
5	25	8	12.15	6	0.72	289.12	296.91	2.70
6	25	9	12.15	6	0.69	272.03	264.98	2.59
7	25	10	8.10	8	1.00	381.42	388.45	1.84
8	25	11	12.15	10	1.10	329.91	325.73	1.27
9	25	7	12.15	4	0.48	286.61	281.84	1.66
10	25	11	8.10	12	1.12	404.20	415.75	2.86
11	30	10	9.72	4	0.22	200.88	200.01	0.43
12	30	11	9.72	4	0.20	194.57	187.06	3.86
13	35	5	9.72	4	0.30	249.35	245.92	1.38
14	35	10	8.10	4	0.30	227.90	233.83	2.60
15	35	8	8.10	4	0.36	250.08	249.81	0.11
16	35	4	8.10	4	0.41	330.87	340.43	2.89
17	35	6	9.72	12	0.76	413.10	425.79	3.07
18	35	11	12.15	6	0.73	261.96	257.18	1.82
19	35	10	12.15	4	0.46	241.17	247.2	2.50
20	25	9	9.72	4	0.16	224.62	225.07	0.20
21	25	10	12.15	8	0.63	268.50	268.05	0.17
22	35	11	12.15	6	0.36	214.74	209.3	2.53
MAPE (%)								1.86

**Figure 6:**
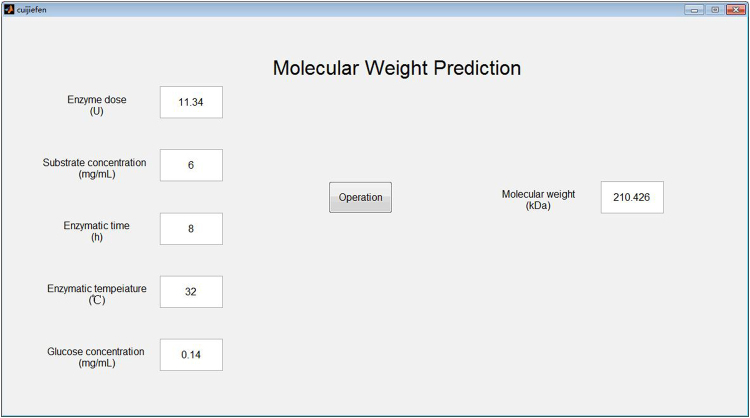
Display of user interface.

When enzymatic hydrolysis condition is changed, the users need to run MATLAB software and modify parameters in the procedure. However, this will make the manipulation complicated. In order to solve the problem, the visual prediction system was developed to create a convenient user interface (Fig. 6). According to Li *et al*.^38^, glucose was one of the hydrolysates. Glucose concentration could reflect the enzymatic hydrolysis process to a certain extent. Consequently, the ANN model could predict molecular weight of the sulfated polysaccharide efficiently by monitoring glucose concentration on-line.

## Conclusions

In the present work, enzymatic products PE3 exhibited the most potent anticoagulant activity. Results indicate that sulfate ester, monosaccharide composition, and molecular weight are important factors for determining the anticoagulant activity in polysaccharides from *E*. *prolifera*. The ANN model was built to simulate the distributions of molecular weight during the complex enzymatic reaction. The developed vision-based ANN model could be an applicable and convenient tool to obtain the desirable polysaccharides with prominent anticoagulant activity. Further efforts along this direction may provide a new dimension for the treatment of thrombosis.

## Materials and Methods

### Materials

*E*. *prolifera* was collected from the coasts of Qingdao, China. Dialysis membrane was purchased from Lvniao (Yantai, China) and molecular weight was cut off at 3500 Da. Dextran standards (50, 80, 150, 270, 410, and 670 kDa), D-glucose, L-rhamnose, D-xylose, and D-glucuronic acid were purchased from Sigma Co. (USA). Activated partial thromboplastin time (APTT), thrombin time (TT), and prothrombin time (PT) reagent kits were purchased from Nanjing Jiancheng Bioengineering Institute. Coagulation analyzer SYSMEX CA 6000 was purchased from TOA Medical Electronics Co. (Kobe, Japan). All other chemicals and reagents were analytical grade.

### Extraction and quantification of PE

The PE was extracted with the improved method as described by Li *et al*.^38^. The milled alga (60 g) was dipped into 40 volumes of tap water, homogenized and extracted at 100 °C for 1 h. The water extraction solution was centrifuged (4000 × g, 10 min), and the supernatant was dipped in 2 times volume of 95% ethanol to remove pigment. After centrifugation, the sediment was collected, and dissolved in distilled water.

### Preparation for degradase for PE (DPE) and Enzyme assay

*Alteromonas* sp. A321 was cultivated and its extracellular supernatant was extracted according to Li *et al*.^38^. Then, the supernatant was brought to 60% (W/V) saturation with (NH_4_)_2_SO_4_. The precipitate collected was dissolved in 20 mM phosphate buffer saline (pH 7.5), yielding DPE. The degradase activity was assayed by the method described by Fenice, Selbmann, Zucconi, and Onofri^39^.

### Preparation of polysaccharides with different molecular weights

The PE [200 mL; 1% (w/v)] was hydrolyzed by the DPE (0.81 U/mL, 20 mL) for 8 h under the conditions of 35 °C, pH 6.5. The 10 mL enzymatic hydrolysate was taken out after two hours and quenched by heating at 100 °C for 5 min. Under the condition of continuous stirring the solution was precipitated by adding 2 times the volume of 95% ethanol. After centrifugation, the sediment was collected, dissolved in distilled water, and dialyzed to give one degraded product named after PE1. The PE2, PE3, and PE4 were gained in the same manner as PE1 except that the hydrolysate was drawn out after 4 h, 6 h, and 8 h, respectively. During the process of preparing the PE4, the supernatant was collected after centrifugation. The supernatant was concentrated, dialyzed, and referred as PE5.

### Characterization

The molecular weights of PE, PE1, PE2, PE3, PE4, and PE5 were determined by using an Agilent 1260 HPLC system (Wilmington, USA) equipped with PL Aquagel-OH 50 column (0.75 cm × 30 cm) and a refractive index detector (RID). The column was eluted with 0.2 M NaNO_3_ and 0.01 M NaH_2_PO_4_ at a flow rate of 0.8 mL/min. The molecular weights of all samples were estimated by referencing the calibration curve made from dextran standards.

The total sugar was determined by phenol-sulfuric acid method using glucose as standard^40^. Sulfate ester content was estimated according to the method described by Therho and Hartiala^41^. The monosaccharide composition was analyzed by using reversed-phase HPLC after pre-column derivatization^42^. The vibrational spectra of different atomic and polar bonds were studied by Fourier transform infrared spectroscopy (FTIR). The FTIR spectra was recorded in the wavelength range of 4000-400 cm^−1^ at a resolution of 4 cm^−1^ using MAGNA-IR 560 E.S.P (Nicolet, USA).

### Anticoagulant activity assays

Activated partial thromboplastin time (APTT), thrombin time (TT), and prothrombin time (PT) assays were carried out according to the method described by Pawlaczyk *et al*.^43^ Saline and the unfractionated heparin were used as negative and positive controls, respectively. All clotting assays were performed with three individual replicates to estimate the mean clotting time. The anticoagulant activity was expressed as the clotting time.

### Experimental design

Enzymatic temperature (25, 30, and 35 °C), enzymatic time (1, 2, 3, 4, 5, 6, 7, 8, 9, 10, and 11 h), substrate concentration (4, 6, 8, 10, and 12 mg/mL), and enzyme dose (8.10, 9.72, and 12.15 U) were considered according to the situation of enzymolysis. In order to construct and train the ANN model, two hundred and thirty-one experimental groups were designed referring to the uniform orthogonal design method.

### ANN modeling

The training of ANN was accomplished by adapting the strengths or weights of the connections among the input, intermediate and output neurons, which were capable of storing memory and information. By achieving the learning ability, ANN produced the desired responses according to the given decision variables^44^.

**Figure 7:**
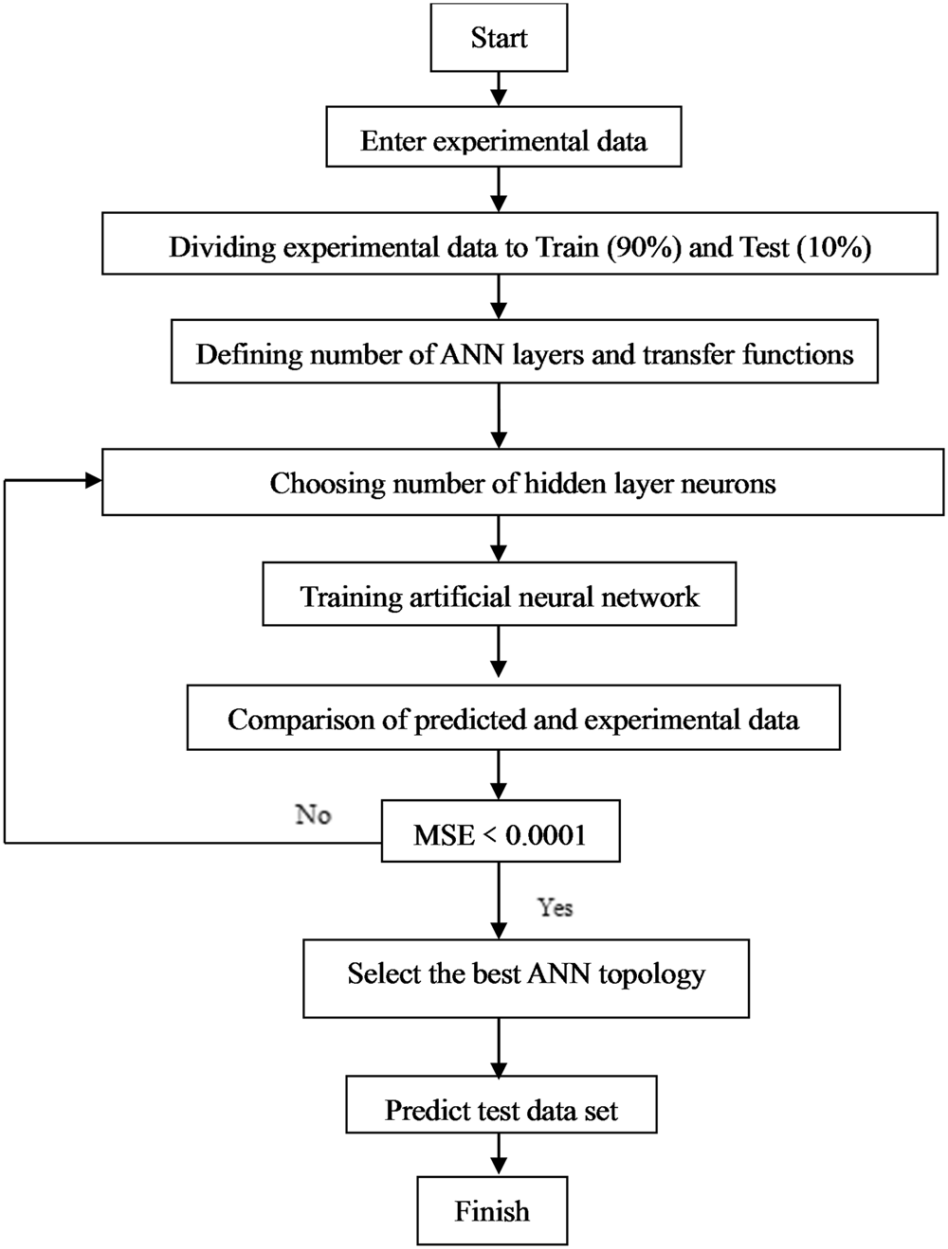
Flowchart of the optimal neural network.

In this study, the ANN model was developed using the neural network toolbox in MATLAB version 8.1. The 231 experimental data points were employed to build the ANN model. They were divided into training and testing sets with a ratio of 90% and 10%, respectively. Flowchart of the optimal neural network is presented in Fig. 7. The equation below was used for data normalization:

$${{\rm{X}}}_{{\rm{i}}}=({\rm{X}}-{{\rm{X}}}_{{\rm{\min }}})/({{\rm{X}}}_{{\rm{\max }}}-{{\rm{X}}}_{{\rm{\min }}})$$

where X_i_, X, X_min_, and X_max_ represented the normalized value, actual value, minimum value, and maximum value, respectively.

The BP network and Levenberg-Marquardt optimization algorithm was applied in the training process. The MSE between the output values and targeted values are calculated and returned back to the hidden layer. Weights between inputs and hidden layer are also calculated, which are updated between the hidden layer and the output layer.

### Statistical analysis

All experiments were repeated in triplicate, and the data were reported as mean ± standard deviation and evaluated by one-way ANOVA. Statistical analysis was performed using MATLAB mathematical software (version 8.1).
